# Supplementary material for: Identification of photoperiod-induced specific miRNAs in the adrenal glands of Sunite sheep (Ovis aries)
Source: Front Vet Sci. 2022 Jul 22;9:888207. doi: 10.3389/fvets.2022.888207 (PMC9354845; doi:10.3389/fvets.2022.888207)
Supplement: Supplementary file 1 [file Data_Sheet_1.ZIP › Supplementary file/miRDeep2 file.zip!/miRDeep2 file/Novel_Structure/SP42A3/Novel_204.pdf]

Provisional ID	:	NC_019471.2_21113
Score total	:	6.4
Score for star read(s)	:	3.9
Score for read counts	:	-0.8
Score for mfe	:	1.8
Score for randfold	:	1.6
Score for cons. seed	:	
Total read count	:	10
Mature read count	:	9
Loop read count	:	0
Star read count	:	1

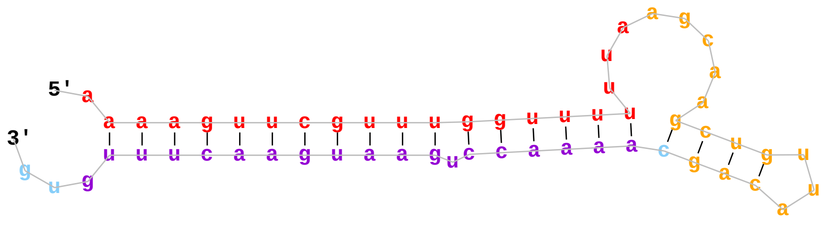

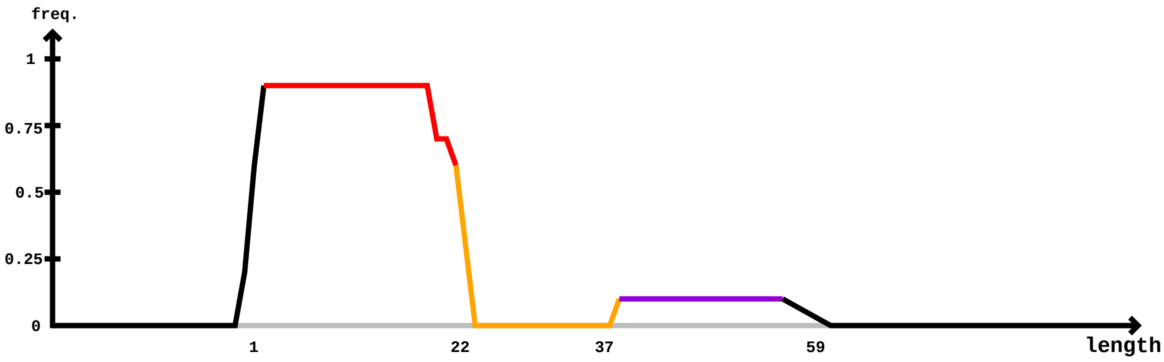

Mature **Star**

```
5'- auacacuacugggcuggccaaaaaguucguuugguuuuuuaagcaagcuguuacagcaaaaccugaaugaacuuuguggccaacccaagacucuguucccacaggcagaca   -3'    obs
    auacacuacugggcuggccaaaaaguucguuugguuuuuuaagcaagcuguuacagcaaaaccugaaugaacuuuguggccaacccaagacucuguucccacaggcagaca          exp
    .........((((.((((((.((((((((((((((((........((((...))))))))))).))))))))))).)))))).))))....((((((......))))))..   reads    mm    sample
    ..................caaaaaguucguuuggGuuu.........................................................................   2        1     seq
    ...................aaaaaguucguuugguuuuuu.......................................................................   1        0     seq
    ...................aaaaaguucguuuggGuuuuua......................................................................   3        1     seq
    ....................aaaaguucguuuggGuuuuua......................................................................   3        1     seq
    ........................................................aaaaccugaaugaacuuuC...................................   1        1     seq
```
